# Supplementary material for: Integrating AAA Care Coordinators in Primary Care for Dementia Support: Implementation Challenges and Lessons Learned
Source: Int J Environ Res Public Health. 2025 Mar 26;22(4):506. doi: 10.3390/ijerph22040506 (PMC12026521; doi:10.3390/ijerph22040506)
Supplement: Supplementary file 1 [file ijerph-22-00506-s001.zip › ijerph-3412443-supplementary.pdf]

Table S1: Data Fields Completed in Area Agency on Aging Care Coordinator Spreadsheet
This table defines various variables used in tracking patient interactions and care coordinator services, including information such as patient demographics, medical history, contact with patients and caregivers, referral details, and notes for future follow-up.

Variable Name	Definition
Date	Date of care manager service
Zip code	Patient residential ZIP code from medical chart
Birth year	Year from patient birth date recorded in medical chart
AD on file	1 indicates that the patient has an advanced directive (AD) on file. 0 indicates not in medical chart. Do Not Resuscitate (DNR) orders do not count; living wills and Indiana advanced directive or PREPARE forms do count.
Dementia Dx	1 indicates that a dementia diagnostic code is listed in the problems identification section of the medical chart. 0 means that the patient does not contain a dementia diagnosis in the medical history.
Fall risk	1 indicates that a fall screen was conducted and fall risk was identified as a problem in the medical chart. 0 means that the patient does not contain a positive fall risk note in the medical history.
EPIC referral	1 indicates that the AAA care manager received a referral from a provider via EPIC. 0 indicates that there was no electronic referral associated with the service (either new interaction with previously referred or care manager was notified outside of EPIC).
Patient contact	1 indicates that the AAA care manager was able to speak with the patient. 0 means the AAA care manager did not speak with the patient.
Caregiver contact	1 indicates that the AAA care manager was able to speak with the caregiver. 0 means the AAA care manager did not speak with the caregiver.
First interaction	1 means this service represents the first time the AAA care manager has spoken with the patient or caregiver.
Referral Option 1	See Table S2 for available options. A blank was used to indicate no support or referral services were provided.
Referral Type 1	Automatically populated based on referral option selected; see Table S2 for the referral type category associated with the selected option.
Referral Option 2	See Table S2 for available options. A blank was used to indicate no support or referral services were provided.
Referral Type 2	Automatically populated based on referral option selected; see Table S2 for the referral type category associated with the selected option.
Referral 3	See Table S2 for available options. A blank was used to indicate no support or referral services were provided.
Referral Type 3	Automatically populated based on referral option selected; see Table S2 for the referral type category associated with the selected option.
Referral 4	See Table S2 for available options. A blank was used to indicate no support or referral services were provided.
Referral Type 4	Automatically populated based on referral option selected; see Table S2 for the referral type category associated with the selected option.

Referral 5	See Table S2 for available options. A blank was used to indicate no support or referral services were provided.
Referral Type 5	Automatically populated based on referral option selected; see Table S2 for the referral type category associated with the selected option.
Notes	Space for the AAA care manager to note follow-up needed, important dates, and/or anything that would be helpful to remember should the care coordinator interact with the patient in the future.

AAA care manager stands for Area Agency on Aging Care Manager.

Table S2: AAA Care Coordinator Referral Options and Referral Type Categories
This table outlines a variety of referral and service options available for AAA Care Coordinators to select to record patient services. Each option was categorized by referral type to support aggregation across the 4Ms of age-friendly care (i.e., What Matters, Mentation, High Risk Medication, and Mobility) and the major health-related social needs (e.g., healthcare access, transportation, etc.) addressed.

Referral/Service Option Available to AAA Care Manager to Select	Analytic Coding
ACP – PREPARE	What Matters
ACP – Conversation	What Matters
ACP – Advance Directive Information	What Matters
ACP - Notarize	What Matters
Adult Day Services	What Matters (Respite)
Medication Services	Medication
Mental Health Resources	Mentation
Alzheimer's Association Direct Referral	Mentation
Alzheimer's Association Information	Mentation
Incontinence Products	Mobility
AAA Activity Center	Mobility
Fall Risk Education and Information	Mobility
Matter of Balance Referral	Mobility
Chronic Disease Education and Information	Mobility
Chronic Disease Self-Management Program Referral	Mobility
Area Agency on Aging Referral	Health Related Social Need
Literacy Referral	Health Related Social Need
Job Placement Referral (AARP)	Health Related Social Need
Legal Assistance	Health Related Social Need
Clothing Bank	Health Related Social Need
Blind Association	Health Related Social Need
Interpreter	Health Related Social Need
Technology Assistance Program Referral	Health Related Social Need
Government Funded Cell Phone	Health Related Social Need
Closed Captioned Phone	Health Related Social Need

Extra Help Application	Health Related Social Need
Food Stamps Application	Health Related Social Need – Food Assistance
Financial Assistance	Health Related Social Need – Financial Assistance
AAA Meal Site	Health Related Social Need – Food Assistance
Meals on Wheels	Health Related Social Need – Food Assistance
Food Pantry	Health Related Social Need – Food Assistance
Home Health Services	Health Related Social Need – Healthcare Access
Hospice	Health Related Social Need – Healthcare Access
Palliative	Health Related Social Need – Healthcare Access
Medical Equipment	Health Related Social Need – Healthcare Access
Medicare Referral	Health Related Social Need – Healthcare Access
Medicaid Application	Health Related Social Need – Healthcare Access
Housing Options	Health Related Social Need – Housing/Safety
Adult Protective Services	Health Related Social Need – Safety
Transportation	Health Related Social Need – Transportation

ACP stands for Advanced Care Planning, while AAA refers to the Area Agency on Aging.
